# Acute respiratory distress syndrome heterogeneity and the septic ARDS subgroup

**DOI:** 10.3389/fimmu.2023.1277161

**Published:** 2023-11-14

**Authors:** Huikang Xu, Shiying Sheng, Weiwei Luo, Xiaofang Xu, Zhaocai Zhang

**Affiliations:** ^1^ Department of Critical Care Medicine, The Second Affiliated Hospital, Zhejiang University School of Medicine, Hangzhou, Zhejiang, China; ^2^ Key Laboratory of the Diagnosis and Treatment for Severe Trauma and Burn of Zhejiang Province, Hangzhou, China; ^3^ Zhejiang Province Clinical Research Center for Emergency and Critical Care Medicine, Hangzhou, China

**Keywords:** acute respiratory distress syndrome (ARDS), sepsis, heterogeneity, immune system, precision therapy

## Abstract

Acute respiratory distress syndrome (ARDS) is an acute diffuse inflammatory lung injury characterized by the damage of alveolar epithelial cells and pulmonary capillary endothelial cells. It is mainly manifested by non-cardiogenic pulmonary edema, resulting from intrapulmonary and extrapulmonary risk factors. ARDS is often accompanied by immune system disturbance, both locally in the lungs and systemically. As a common heterogeneous disease in critical care medicine, researchers are often faced with the failure of clinical trials. Latent class analysis had been used to compensate for poor outcomes and found that targeted treatment after subgrouping contribute to ARDS therapy. The subphenotype of ARDS caused by sepsis has garnered attention due to its refractory nature and detrimental consequences. Sepsis stands as the most predominant extrapulmonary cause of ARDS, accounting for approximately 32% of ARDS cases. Studies indicate that sepsis-induced ARDS tends to be more severe than ARDS caused by other factors, leading to poorer prognosis and higher mortality rate. This comprehensive review delves into the immunological mechanisms of sepsis-ARDS, the heterogeneity of ARDS and existing research on targeted treatments, aiming to providing mechanism understanding and exploring ideas for accurate treatment of ARDS or sepsis-ARDS.

## 1 Introduction

Acute respiratory distress syndrome (ARDS) is a diffuse lung injury caused by intrapulmonary and extrapulmonary factors in a short period, histologically characterized by diffuse alveolar damage, including pulmonary edema, hyaline membrane formation, alveolar hemorrhage and inflammation (1–3). ARDS is a heterogeneous syndrome, and its heterogeneity is mainly manifested in physiology, imaging findings, etiology, onset time, biomarker and gene differences, etc. Among the subtypes of ARDS, septic ARDS is a noteworthy and complex one. Sepsis is a major risk factor for ARDS, accounting for 32% of ARDS etiology (4). The prognosis of patients with sepsis-induced ARDS is worse than that of patients with sepsis or ARDS alone. This subgroup of ARDS usually faces a higher mortality rate and a lower success rate of extubation, which has received more attention in clinical practice (5, 6).

Sepsis is a systemic inflammatory reaction caused by a maladjusted response to infection induced by pathogenic microorganisms (7). The overwhelming inflammatory molecules, released due to infection in sepsis, not only flow along the systemic blood circulation, but also form a cascaded and amplified network with the participation of the nervous, endocrine and immune system (8). Sepsis is a common disease in intensive care units (ICU). Research had revealed that sepsis has an incidence of 437/100,000 on a global scale, with a case fatality rate of 17% (9). Another two-month prospective observational cohort study in China mainland showed that 54.8% of ICU patients would progress from severe sepsis to a comorbidity of sepsis-ARDS, and the proportion of non-surviving patients in sepsis-ARDS reached 71.0% (10). The cytokine storm caused by sepsis can bring about either direct damage to the lung epithelium or indirect damage to the lung endothelium (11, 12). Due to large individual differences, it is difficult to obtain reliable results in clinical trials of ARDS, and some treatments are only effective in a small portion of patients (13, 14). Therefore, it is crucial to explore the heterogeneity of ARDS and the immunological mechanism behind it for the effective therapy. This review mainly introduces the heterogeneity of ARDS, the related precision medicine and the immunological mechanism of sepsis-ARDS, hoping to provide some assistance and guidance for researchers in the study of the heterogeneity of ARDS and the treatment of sepsis-induced ARDS.

## 2 ARDS heterogeneity

The clinical, physiological, biological and imaging manifestations of ARDS are highly heterogeneous. The etiological factors of ARDS can be divided into two types: intrapulmonary and extrapulmonary. The intrapulmonary factors, such as pneumonia and mechanical ventilation, primarily inflict damage on the alveolar epithelium, whereas extrapulmonary factors like sepsis and acute severe pancreatitis initially impair the endothelium and subsequently result in pulmonary edema (15). The 1994 American-European Consensus Conference(AECC) had suggested that this heterogeneity would greatly hinder the research on the treatment of ARDS (16). The Consensus Conference in 1994 laid the foundation for a better understanding of ARDS, and the Berlin Definition published in 2012 defined ARDS in more detail and had classified ARDS patients according to the PaO_2_/FiO_2_ level (17). With the proposal of the Berlin definition, some researchers conducted autopsies on 712 patients with ARDS according to the Berlin definition, which clarified the high sensitivity and low specificity of the Berlin definition, and researchers confirmed the pathological heterogeneity of ARDS as well (18). Recently, the new definition of ARDS has been proposed based on the original Berlin definition, which has been fine-tuned and modified. For example, the pulse oxygen saturation SpO2/FiO2 is allowed to replace PaO2/FiO2 in the classification criteria (3). These definitions reflect an evolving understanding of the heterogeneity of ARDS as a complex syndrome that needs to be treated differently, on a case-by-case basis, as compared with the previous defining as a whole. The specific changes in the new definition relative to the Berlin definition are shown in **Table 1**.

**Table 1:** Berlin definition and new global definition of ARDS (3, 17).

	Berlin definition	New global definition
Disease onset	Existing clinical injury or exacerbation of new or existing respiratory symptoms occurred within one week	The onset of COVID-19 cases may be prolonged. HFNO was included to capture those with a slow course of disease.
Pulmonary imaging findings	X-ray or CT scans show a bilateral dense shadow of the lung that cannot be explained by effusion, lobar pneumonia, atelectasis, or nodules	In addition to bilateral opacities on chest radiography and CT, the absence of ventilation identified by ultrasound can be also used as the criterion.
Oxygenation index	Mild: 200 mmHg<PaO_2_/FiO_2_ ≤ 300 mmHg with PEEP/CPAP≥5cmH_2_O Moderate: 100 mmHg< PaO_2_/FiO_2_ ≤ 200 mmHg with PEEP≥5cmH_2_O Severe: PaO_2_/FiO_2_ ≤ 100 mmHg with PEEP≥5cmH_2_O	In addition to PaO2/FiO2, SpO2/FiO2 can also be used for severity assessment when SpO2 ≤ 97%. A new category of non-intubated ARDS was created for patients with HFNO≥30L/min who met the criteria for ARDS, and PaO2/FiO2 and PEEP were no longer mandated as limited-condition criteria.

PAWP, Pulmonary artery wedge pressure; PEEP, positive end-expiratory pressure; CPAP, continuous positive airway pressure; FiO2, Fraction of inspired oxygen; PaO2, Partial pressure of arterial oxygen; HFNO, High flow nasal oxygen; SpO2, Pulse oximetric oxygen saturation.

Many clinical trials that treated patients with ARDS as a unified group have failed (19). And latent class analysis of failed clinical trials briefly came into focus (20–22). Due to the failure of a large number of clinical trials, more individuals are paying attention to the subgrouping of ARDS to obtain the meaningful experimental results (**Table 2**). With this subgrouping, one hopes to harvest different and beneficial clinical trial results in the near future.

**Table 2:** ARDS heterogeneity summary.

Derivation of ARDS heterogeneity	Main observation target	Manifestations	Targeted treatment
Physiology	PaO_2_/FiO_2_	Mild; moderate; severe	Prone ventilation and neuromuscular blockers for severe ARDS (23, 24)
Imaging	CT imaging findings	Diffuse ARDS; lobular ARDS	Only CT findings of ARDS with diffuse lung injury benefits from PEEP (25)
Onset time	ICU admission to 48 hours	Early-onset ARDS; late-onset ARDS	Early-onset ARDS had a better response to PEEP (26)
Biomarker	Biomarker expression level	Hyperinflammatory; hypoinflammatory	Simvastatin was more effective for treatment of hyperinflammatory ARDS than hypoinflammatory ARDS (27).
Etiology	Initial damaged organs	Pulmonary ARDS; extrapulmonary ARDS	Extrapulmonary ARDS responded well to prone ventilation and PEEP (28, 29).
Genetic susceptibility	Key gene variants	Ang-2; IL-1β; IL1RN	Targeted therapy for genes (30)

### 2.1 Physiologically subgrouping

ARDS is a multi-etiology disease, and different etiologies will bring physiological variability. The grouping of ARDS from a physiological perspective will be a key point to figure out its heterogeneity. According to the Berlin definition in 2012, ARDS was divided into mild, moderate and severe types based on PaO2/FiO2 ratio (17). Patients with severe ARDS (PaO_2_/FiO_2_ ≤ 150mmHg) were selected for randomized controlled trials of prone ventilation and neuromuscular blockers as a research object. Both trials shown that these two treatments can significantly improve 90-day survival (23, 24). The definition of severe ARDS here differs from the Berlin definition, in which PaO_2_/FiO_2_ ≤ 100mmHg, possibly because the two randomized trials were designed before the declaration of Berlin definition. In a multicenter prospective study, patients with increased lung dead-space tended to have a higher risk of mortality (31). The dead-space fraction is an independent risk factor for ARDS patients (31). Perhaps in future clinical trials, the lung dead-space fraction will be included in the grouping criteria.

### 2.2 Imaging findings variety

In the progression of ARDS, the shadow range and lesion range in the imaging results of patients are varied, which brings a certain degree of hindrance to the diagnosis and treatment. Pulmonary ARDS is usually characterized by extensive ground-glass opacification and asymmetric lung consolidation, while extrapulmonary ARDS is characterized by symmetrical ground-glass opacification distributed in perihilar regions (32). Desai et al. tend to distinguish pulmonary ARDS and extrapulmonary ARDS by the typical or atypical CT manifestations. The typical CT manifestations are characterized by extensive and intense opacification of the dependent parenchyma, whereas the atypical CT manifestations involve more extensive and intense opacification of the non-dependent parenchyma. Researchers have observed that extrapulmonary ARDS often exhibit typical CT features (33). Some studies have divided the ARDS into diffuse ARDS and lobular ARDS by CT findings, and found that the two types have distinct responses to positive end expiratory pressure (PEEP) (25). In patients with diffuse ARDS, PEEP brought about significant alveolar dilation, while in patients with lobar ARDS, only mild alveolar dilation was induced by PEEP (25). These imaging findings have guiding implications for clinical management of ARDS. Radiological features are the most intuitive means to detect inflammatory infiltration in the lung, which can usually provide rapid guidance to clinicians when fighting against the illness.

### 2.3 Heterogeneity in the onset time of ARDS

The Berlin definition sets the onset of ARDS as within one week of the occurrence or exacerbation of the underlying injury (17). In 2009, Liao et al. took 48 hours as the cut-off line and divided patients admitted to ICU into early-onset(<48h after ICU admission) and late-onset ARDS(>48h after ICU admission), and they found that these two have different prognoses and mortality (34). Data from a multicenter observational study showed that patients with early-onset ARDS had higher scores in both Simplified Acute Physiology Score (SAPS) II and initial Sequential Organ Failure Assessment(SOFA), whereas those with late-onset ARDS had longer ICU and hospital stays (35). Therefore, the onset time of ARDS is also a very important heterogeneous factor affecting the outcome of patients. The classification of ARDS according to the onset time after admission can sometimes predict the possible clinical manifestations and thus make treatment more favorable. For example, studies have found that early-onset ARDS is associated with severe hemorrhagic shock, while late-onset ARDS is often characterized by pneumonia and multiple organ damage (26). Moreover, early-onset ARDS responds better to protective mechanical ventilation (26). The different onset time of ARDS can bring about protein biomarker diversity, such as receptor for advanced glycation end product (RAGE)s and Angiopoietin (Ang)-2, markers of alveolar capillary barrier injury, which are only elevated in early-onset ARDS (36). The variations in pathological manifestations, response to therapy, and levels of circulating proteins underscore the importance of classifying ARDS based on its onset time.

### 2.4 Biomarker heterogeneity

Biomarkers of ARDS can not only indicate the pathogenesis and progression of the disease, but also conduce to identify ARDS subgroups and provide targeted treatment (37–39). RAGE is mainly distributed in the basal surface of alveolar type 1(AT1) epithelial cells, and it engages in innate immunity and important alveolar inflammatory pathways (40, 41). Soluble RAGEs, a marker of type I alveolar epithelial cell injury, produced by cleavage of full-length RAGE, are increased only in the early stages of ARDS (39, 42). The randomized controlled trial of a single-center study found a significant difference in soluble RAGE level between direct ARDS and indirect ARDS (43). In addition to RAGE, the levels of Surfactant protein D (SP-D), IL-6, IL-8 and von Willebrand factor (vWF) were also significantly different between these two groups (43). In an observational study, patients with non-focal ARDS had higher plasma levels of soluble RAGE (44).

In ARDS, RAGE reflects alveolar epithelial damage, whereas Ang-2 is a marker of endothelial cell activation and increased capillary permeability. In a randomized controlled trial, plasma Ang-2 level was proved to predict the prognosis and mortality of patients with ARDS, and high levels of Ang-2 usually suggest poor prognosis (45). In critically ill patients, the early elevation of Ang-2 can predict the impending occurrence of acute lung injury (ALI) (46). Ang-2 in ARDS could reflect endothelial injury, which is common in extrapulmonary ARDS.

Another important plasma marker associated with ARDS severity is IL-1 receptor antagonist (IL-1RA), a naturally occurring substance secreted by monocytes, which can bind to the IL-1 receptor without initiating transcription and thus exert an anti-inflammatory effect (47). Due to the anti-inflammatory effect of IL-1RA, it is universally accepted that IL-1RA may play a protective role in ARDS and sepsis. In the 1994 randomized controlled trial, IL-1RA was found to have no statistically significant therapeutic effect in patients with sepsis (48). Nevertheless, in the *post hoc* analysis, IL-1RA improved survival in patients with severe sepsis who had organ failure or a high predicted mortality rate (>24%) (48). In another randomized controlled trial in sepsis (including septic ARDS), no clear therapeutic benefit of IL-1RA was found either (49). In the upgraded clinical trial, when sepsis patients (including ARDS patients) were grouped according to the plasma level of IL-1RA at enrollment, the high IL-1RA level group tended to obtain survival benefits (50). The ILRN variant rs315952C present in subjects of European ancestry increases plasma levels of ILR1A and patients with the variation had higher survival rate in patients with septic shock (50). Additionally, endothelin serves as a crucial biomarker for sepsis-induced ARDS, and its production actively contributes to the progression of organ dysfunction in sepsis, including ARDS (51). In the context of sepsis-induced ARDS, free fatty acid (FFA)s/non-esterified fatty acid (NEFA)s emerge as another crucial substances. Free fatty acid serum C18 can be used as an important predictor of ARDS development (52). In animal experiments, reducing plasma non-esterified fatty acid (NEFA) levels has been shown to decrease the incidence of lung injury (53).

Beyond that, other markers can also be used to observe the heterogeneity of ARDS including thrombomodulin, TNF-1 receptor and so on (54, 55). Latent class analysis after randomized controlled trials found that when ARDS patients were divided into hyperinflammatory and hypoinflammatory subgroups according to the expression levels of inflammatory cytokines, the clinical manifestations and prognoses of the two subgroups were widely different (56). In this subgrouping, ARDS patients were regrouped according to inflammatory biomarkers, and patients with hyperinflammatory ARDS had a higher incidence of shock and mortality rate, and they response better to PEEP (20). A suitable and stable biomarker not only enhance comprehension of the disease progression, pathogenesis and possible prognosis of ARDS, but also facilitate physicians to make appropriate treatment decisions for different subtypes of ARDS.

### 2.5 Etiological diversity of ARDS

The etiology of ARDS is diverse and variable, including pneumonia, aspiration of stomach contents, sepsis, acute severe pancreatitis, extensive burn, etc. Pneumonia and non-pulmonary sepsis are the most common risk factors for ARDS (6). A retrospective cohort study of microbial-positive pneumonia found that bacterial infection was the leading cause of pneumonia, followed by fungal, viral, and mixed pathogen infections (57). However, the incidence of lung injury induced by bacterial pneumonia is lower than the latter (57). For patients with sepsis, the incidence of ALI caused by gram-positive and gram-negative bacterial infections was similar (57). For patients with viral infection, studies had pointed out that influenza A virus is the main viral cause of ARDS in adults (58). Compared with H1N1 influenza virus group, which mainly causes mild and moderate ARDS, H7N9 influenza virus infection has a high risk of severe ARDS (59, 60). In addition, the infection of severe acute respiratory syndrome coronavirus 2 (SARS‐CoV‐2) could also lead to ARDS, namely the severe COVID‐19,which brings about significant lung injury by binding to the ACE2 receptor (61). Similar to sepsis induced ARDS, COVID-19 are characterized by coagulation dysfunction, which is prone to form pulmonary thrombosis and lung dysfunction (61). Vascular enlargement is a specific imaging finding of lung injury caused by COVID-19, differing from the etiology of other pathogens (61, 62).

Different etiology often leads to different clinical manifestations and mechanisms. As early as 1998, researchers had found that ARDS caused by pneumonia and extrapulmonary factors (trauma, peritonitis, shock, intestinal infection, etc.) has different pathologies and distinct responses to PEEP (63). Since then, ARDS had been divided into pulmonary and extrapulmonary subgroups according to etiology, and a series of studies have been conducted on ARDS according to this classification. Compared with indirect injury (extrapulmonary ARDS), patients with direct injury (pulmonary ARDS) would have higher lung compliance and less responsiveness to PEEP (64). Alveolar epithelial injury, alveolar collapse and fibrin deposition mostly occur in pulmonary ARDS, while extrapulmonary ARDS mainly causes vascular endothelial injury (15, 65). Wide differences in imaging manifestations and biomarkers between pulmonary ARDS and extrapulmonary ARDS arouse extensive concern (32, 43). Additionally, several studies had shown that the mortality of those two are not the same. The mortality rate of extrapulmonary ARDS was slightly higher than that of pulmonary ARDS, but the difference was not significant (66, 67). Various etiologies of ARDS often have different outcomes and pathophysiological mechanisms. Subgrouping of ARDS by etiologies can predict the likely progression of the disease and prevent the occurrence of serious conditions.

In clinical practice, the incidence of sepsis or ARDS is higher than that of sepsis-induced ARDS, but the prognosis of sepsis-induced ARDS is worse (68, 69). Sepsis-related ARDS has a lower PaO2/FiO2 ratio, more obvious dyspnea, longer recovery time, and lower success rate of extubation than non-sepsis-related ARDS (5, 6, 70). According to previous studies, sepsis is the main cause of ARDS, accounting for 31% of the etiology of ARDS, and ARDS is a serious and destructive complication of sepsis (4). Extreme hypoxia approximately account for 38.2% of mortality in ICU (5). Hence, sepsis-induced ARDS is a notable group, and further research on this subgroup may immensely reduce the mortality of respiratory causes in ICU.

### 2.6 Genetic variability

When studying the genetic characteristics of ARDS, it is arduous to obtain a family pedigree of ARDS patients, because the disease is often secondary to other serious systemic diseases and most cases are sporadic (71). However, researchers can study the association between individual genetic variants about ARDS risk factors. Studies found that the single nucleotide polymorphism (SNP) rs1190286 in the POPDC3 gene was associated with a reduced risk of ARDS, and the -308A allele of TNF was associated with increased mortality of ARDS (72, 73). The SNP rs315952C of IL1RN can increase the plasma level of IL1RA and reduce the risk of ARDS (74). In the study of the causal relationship between genes and diseases, Mendelian randomization analysis is commonly used (75, 76). Through the analysis of haplotypes of IL-1β, it was found that plasma level of IL-1β can affect the 90-day mortality of sepsis (77). As a key marker of endothelial activation and permeability, Ang-2 gene variation is also crucial for the disease risk prediction of ARDS. Studies have found that gene variation in Ang-2 may lead to an increased risk of ARDS, such as the variant T allele of one tSNP (rs2515475) and haplotype TT in block 2 containing the T allele (78). The association between the development of ARDS and this SNP was found to be dependent on the linkage disequilibrium between rs2515475 and rs2959811, primarily occurring in subjects of extrapulmonary injury (78). The primary reason for the impact of Ang-2 gene variation on ARDS occurrence lies in its association with pulmonary vascular permeability, leading to an increased likelihood of pulmonary edema fluid accumulation when extrapulmonary injury affects the lungs (79). Furthermore, it has been demonstrated that genetic variations in the Ang-2 gene can alter its expression (80). The plasma level of Ang-2 can serve as a reliable prognostic and mortality predictor for patients with sepsis and ARDS (81). Genetic polymorphisms contributing to ARDS occurrence are generally relevant to innate immunity, surfactant function, oxidative stress, and capillary endothelium (71). Genetic variants correlated to the pathogenesis of ARDS are the root of the heterogeneity of ARDS. Candidate genes can be obtained by microarray gene expression analysis, genome-wide association analysis, RNA sequencing and so on. Studies of candidate genes can contribute to further identify the causal genes in ARDS pathogenesis.

### 2.7 Individual variation

Individuals with underlying diseases and diverse personal characteristics or living habits also exert a certain influence on the occurrence and progression of ARDS. Various studies have demonstrated that patients suffering from diabetes, severe obesity, hypertension and cardiovascular disease are more prone to experiencing unfavorable outcomes following infection with COVID-19 (82–84). Diabetic and obese individuals typically exhibit compromised innate and adaptive immunity, along with a persistent state of chronic low-grade inflammation (85). In a regression cohort study, advanced age was found to be significantly associated with an increased risk of mortality, and patients who developed ARDS as a complication of COVID-19 were observed to have a higher prevalence of hypertension and diabetes (86). Additionally, lifestyle behaviors such as smoking and drinking can affect ARDS progression and prognosis. A 15-year cohort study revealed a dose-response correlation between smoking and the development of ARDS, whereas no such association was observed among individuals who consumed alcohol (87). Studies found that exposure to cigarette smoke results in an increased influx of neutrophils and induces damage to the alveolar epithelium (88). However, the meta-analysis of observational studies conducted between 1985 and 2015 by Simon et al. revealed a significant association between alcohol consumption and an increased risk of ARDS (89). This may be attributed to the depletion of glutathione resulting from chronic alcohol exposure, which subsequently leads to dysfunction of alveolar epithelium and failure in clearing alveolar fluid (90). The presence of significant inter-individual variations and diverse risk factors among patients often exerts an impact on the onset and progression of ARDS, thereby posing challenges to clinical diagnosis and treatment.

## 3 Pathogenesis of septic ARDS

**Figure 1:**
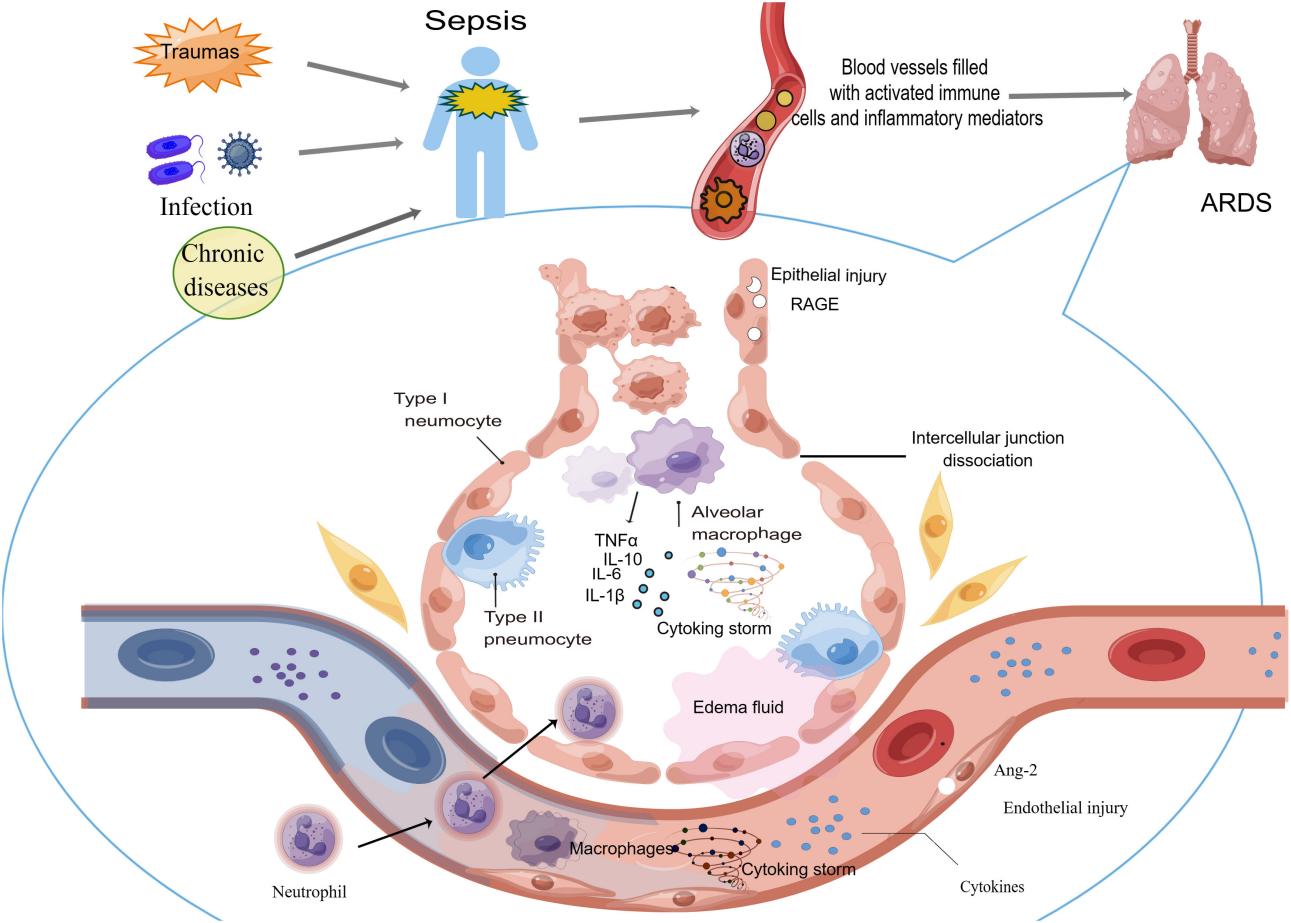
Sepsis can cause ARDS through circulating immune cells and cytokines. After the host undergoes the attack causing sepsis, circulating immune cells are activated and abundant inflammatory mediators are released in the blood. These cells and cytokines injure the vascular endothelium systemically, including the capillary endothelium of the lung. When the endothelium of pulmonary capillaries is damaged, activated immune cells migrating into the lung aggravate the pulmonary inflammatory response and eventually lead to ARDS occurrence.

Sepsis is the most common pathogenic factor of ARDS, which can cause ARDS in less than a week, and the prognosis of those patients is poor (91). ARDS, a common clinical critical disease, has a mortality rate as high as 40%, and septic ARDS has a worse prognosis and higher mortality (91). The underlying mechanism of sepsis-induced ARDS is very complex, and the general process may be as follows: under the action of injury factors (such as bacterial and viral attacks, traumas, peritonitis and so on), circulating immune cells are activated and inflammatory mediators are overwhelmingly released in the blood, which damages the systemic vascular endothelium, including the capillary endothelium of the lung. When the endothelium of pulmonary capillaries is damaged, it will cause the activation of immune cells in the lung. These cells aggravate the pulmonary inflammatory response and eventually leads to lung injury (**Figure 1**). Septic ARDS are mainly histologically manifested in five aspects: epithelial and endothelial injury, various cell death, oxidative stress, microcirculation disorder and pulmonary edema.

### 3.1 Vascular endothelial and alveolar epithelial injury

The development of sepsis-induced ARDS can be attributed to both direct and indirect lung injury (11). The air-exchange function of the alveoli is maintained by the alveolar-capillary barrier, which is mainly composed of three parts, including the epithelial cell layer, the microvascular endothelial cell layer, and the interstitial space between the epithelium and the endothelium. The destruction of the barrier will disrupt the ventilation/perfusion ratio, leading to lung injury and dysfunction (92). Vascular endothelial integrity is synergistically established by vascular endothelial cadherin and the endothelial receptor kinase Tie2, and this process is regulated by vascular endothelial protein tyrosine phosphatase (VE-PTP) (93). Sepsis down-regulates the expression of Angpt-1, Tek, and KDR in systemic tissues at the transcriptional level, and Angpt-1, Tek, KDR corresponds to Ang-1, Tie2, and VEGFR2 or Flk-1.When these proteins are down-regulated, permeability of pulmonary capillary endothelial is increased and then lung injury develops (94).The alveolar epithelium, composed of alveolar type I and type II cells, is primarily responsible for gas exchange and alveolar fluid clearance in the lung. Epithelial cell death is the main feature of alveolar damage in ARDS, which can be caused by bacterial and viral infection, mechanical stretch, hypoxia and other reasons (95).

### 3.2 Cell death

With the progress of scientific research, scientists have realized that the modes of cell death include but are not limited to necrosis and apoptosis necroptosis, pyroptosis, ferroptosis, lysosomal-dependent cell death, autophagy-dependent cell death and so on (96). Apoptosis is the main form of programmed cell death without producing inflammation (97). Excessive apoptosis of cells is critical in the progression of sepsis-induced ARDS. In sepsis-induced lung injury, apoptosis mainly appeared in endothelial rather than epithelial cells (98). Microvascular dysfunction induced by sepsis is closely related to vascular endothelial cell apoptosis, which is mediated by caspases and iNOS/NAPDH oxidase pathways. Besides, some studies had shown that in sepsis-induced ARDS, the degree of neutrophil apoptosis is inversely proportional to the disease severity, and the delay of neutrophil apoptosis will aggravate lung tissue injury (99).

In ARDS, inflammation and cell death interact such that both processes are gradually and automatically amplified, creating a vicious cycle. One study demonstrated that upregulation of IL-1R1promotes alveolar macrophage pyroptosis and worsens lung injury (100). In addition, inflammasomes released from pyroptotic cells can also induce IL-1β activation and enhance inflammatory responses in macrophages (101). Pulmonary endothelial cells also underwent caspase-11-mediated pyroptosis in sepsis-induced lung injury (102). Caspase-11, activated by LPS, can cleave Gasdermin D into polypeptides and form nanopores in the cell membrane, causing pyroptosis and the release of the cytokine IL-1β (103, 104). Extensive lung endothelial cell death, the release of lactate dehydrogenase (LDH) and IL-1β, and the destruction of endothelial cell barrier are important pathological mechanisms of sepsis-induced ARDS.

In inflammation, necrosis of macrophages can be regulated by a receptor-interacting protein kinase (RIPK)1-RIPK3 complex-dependent pathway, namely necroptosis. Necroptosis is driven by necrosome and it alerts the immune system after overwhelming damage to the host, involving cell swelling, membrane rupture, and release of cytoplasmic contents (105). LPS-TLR4 signaling pathway can promote the occurrence of alveolar macrophage necroptosis (106). The recognition of TLRs towards bacteria is closely associated with bacterial cell wall. TLR2 primarily recognizes the cell wall components of Gram-positive bacteria and peptidoglycan from Staphylococcus aureus, while TLR4 plays a pivotal role in LPS signaling transduction from Gram-negative bacteria (107). Necroptosis of endothelial cells and epithelial cells can also exist in sepsis-induced ARDS (108–110). In a mouse model of sepsis, RIPK3 deficiency significantly prolonged the survival of mice, and pretreatment with necrostatin-1 can reduce mortality rate in systemic inflammatory response syndrome (111, 112).

Ferroptosis, as a form of cell death dependent on the regulation of iron and ROS, is also involved in the disease progression of ARDS caused by sepsis. Studies have shown that neutrophil extracellular traps (NET)s can regulate sepsis-induced ARDS by activating ferroptosis in alveolar epithelial cells (113). Ferroptosis exists not only in alveolar epithelial cells, but also in alveolar capillary endothelial cells and macrophages (114–117). Researchers are inclined to investigate targeted drugs that can effectively inhibit the process of ferroptosis, thereby potentially impeding the progression of sepsis-induced ARDS (117–120). In addition, autophagy also plays a role in the process of sepsis-induced lung injury. Autophagy is a cytoprotective process that promotes the degradation and recycling of cellular components, and its activation can alleviate lung injury and inflammation caused by sepsis (121). Dysregulation of autophagy can lead to the aggravation of lung injury, pulmonary fibrosis, chronic obstructive pulmonary disease and other lung diseases (122–125).

### 3.3 Oxidative stress

Oxidative stress generally refers to a state of imbalance between oxidation and antioxidant defense system (126). Oxidation is basically caused by highly oxidizing substances such as ROS and reactive nitrogen radicals (RNS).The production of these substances can not only fight against invading pathogens, but also oxidize or damage DNA, proteins and lipids, inducing gene mutations, protein denaturation and lipid peroxidation (127). Under physiological conditions, the oxidation and reduction of the human body can reach a state of equilibrium. In the pathological situation of ARDS caused by sepsis, plentiful neutrophils flood into lung tissue and alveolar space, and neutrophils contain NADPH oxidase complexes, which are the primary source of ROS (128). Excessive ROS can bring about lung epithelial and endothelial injury. A study showed that instillation of enzymes producing oxygen metabolites in the rat lung alone can cause non-neutrophil-dependent ALI (129). Moreover, oxidative stress can also cause pulmonary capillary endothelial injury by impairing vasodilation, increasing the adhesion of leukocytes and platelets to the blood vessel wall, and improving capillary permeability, which eventually leads to lung injury (130). Nowadays, molecules associated with oxidative stress has been considered a vital and new target for the treatment of septic ARDS disease, and antioxidant therapy is an essential part of the therapy of sepsis (131, 132).

### 3.4 Pulmonary microcirculation disorder

Sepsis is a systemic disease that can affect the microcirculation of the lung through hemodynamic changes and vascular endothelial damage. In sepsis patients, the deformability of erythrocytes reduced and the cell aggregation increased, resulting in microvascular circulation disorders (133, 134). Sepsis can cause the damage of the endothelial and the release of the inflammatory cytokines, leading to the adhesion and aggregation of the immune cells and platelets on endothelial cells, which possibly results in microvascular thrombosis and the occurrence of ARDS. During sepsis, cytokine storms can lead to the overexpression of nitric oxide (NO) synthase on endothelial cells and the synthesis of NO, resulting in vasodilation and adrenergic hyporeactivity, which affects microcirculatory hemodynamics (135). It has been reported that intestinal ischemia can induce a massive accumulation of neutrophils, leading to extensive occlusion of pulmonary arteries, veins, and microvessels (136). Park et al. used customized video-rate laser scanning confocal microscopy and lung imaging windows to show that of in the sepsis-induced ALI, microvascular perfusion in the lung was greatly affected and regulated by Mac-1 in neutrophils (137). The diameter of neutrophils is larger than that of pulmonary capillaries, and neutrophils must deform to enter capillaries, which is known as neutrophil sequestration (138, 139). Neutrophils will form clusters that block capillaries and arterioles, leading to the disorder of microcirculation in the lung and the formation of dead space with ventilation-perfusion mismatch (137). Besides, NETs formed by neutrophils could block capillaries in the lungs (140). The occurrence of pulmonary microcirculation disorders can result in the accumulation of metabolic waste within the lung, leading to the development of pulmonary edema, inflammation, and hypoxia.

### 3.5 Pulmonary edema

The pathological feature of ARDS includes non-cardiogenic pulmonary edema (141), primarily resulting from both alveolar-capillary barrier damage and impaired clearance of edema fluid (142). Moreover, the ability of clearing edematous fluid in ARDS patients was associated with shorter length of ICU stays and reduced mortality (143). The edema fluid of ARDS can be transported from the alveolar cavity to the alveolar interstitium and eliminated by lymphatic drainage, while the alveolar fluid can be transferred to the vasculature for equilibrium via blood circulation (142). During the clearance process, the transport of alveolar fluid is mainly driven by active Na+ transport located in the alveolar epithelium. Na^+^/K^+^-ATPase drives Na^+^ channels to transport Na^+^ to achieve the clearance of alveolar fluid (142). In ARDS patients, the impaired ability of lung to clear edema may be related to the dysfunction of Na^+^/K^+^-ATPase located in alveolar epithelium (144). Studies have found that improving the function of Na^+^/K^+^-ATPase and/or epithelial Na^+^ channels, such as using beta-adrenergic agonists and increasing sodium transport, can improve alveolar fluid clearance (145–147). Adenovirus-mediated transfer of Na^+^/K^+^-ATPase β1 subunit genes can also increase lung fluid clearance (148). When this enzyme is inhibited or the associated ion channels are inhibited, alveolar edematous fluid will be poorly absorbed (149). Hence, exploring the mechanism of edema clearance and targeting the NA+/K+-ATPase holds significant guiding implications for the treatment of ARDS.

## 4 Immunological mechanisms of sepsis-induced ARDS

**Figure 2:**
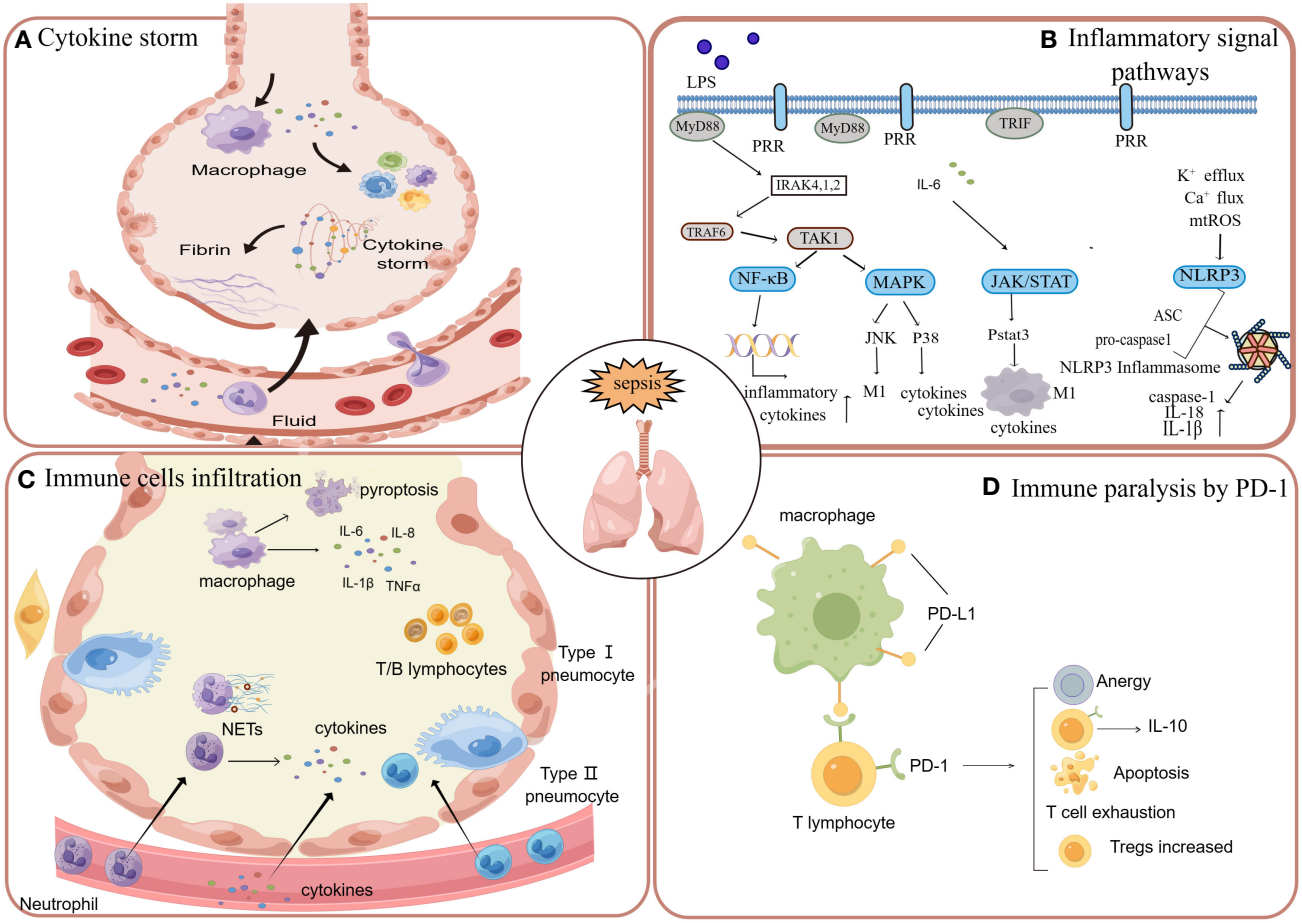
When the systemic system is in a state of sepsis, the inflammation in the circulation will affect various tissues and organs, in which the lung is often affected. **(A)** When the cytokine storm occurs in the circulation, cytokines can enter the lung through the broken endothelium and cause damage to the lung tissue. **(B)** Sepsis induces lung injury through inflammatory signaling pathways, including nuclear factor kappa B (NF-κB), JAK/STAT, and mitogen-activated protein kinase (MAPK). **(C)** In addition to the activation of resident macrophages in the alveoli, neutrophils, macrophages, a few lymphocytes and monocytes can also enter the alveoli through the damaged endothelium, causing inflammation and tissue damage. **(D)** With the massive activation of inflammation caused by sepsis, there will be apoptosis and depletion of immune cells, which is manifested as immune paralysis. The apoptosis, dysfunction and exhaustion of T cells are mainly mediated by the PD-1 pathway.

When pathogens invade the host, the innate immune system responds immediately (150). The pattern recognition receptors (PRRs) can be expressed by both innate immune cells and epithelial cells of the lung (151). PRR can recognize invading pathogens and endogenous molecules through pathogen-associated molecular patterns (PAMP) and damage-associated molecular patterns (DAMP). In this process, immune responses are activated and amplified, which forms cytokine storms. The secretion and release of numerous pro-inflammatory substances can result in vascular endothelial dysfunction, thereby further enhancing pulmonary microvascular permeability, which ultimately facilitates the occurrence and progression of ARDS (152, 153). Following lung injury, innate immune cells such as neutrophils and monocytes are recruited to the alveolar space, leading to subsequent damage of the alveolar epithelium and endothelium. Consequently, a significant accumulation of edema fluid occurs in both the alveoli and interstitium (95, 154). During the pathogenetic process of sepsis-induced ARDS, the host is not always in a state of hyperimmunity. Due to the complex negative feedback of the immune system and immune exhaustion, it will also show a state of immune paralysis in the later stage of the disease (155). The immune system exhibits diminished or absent responsiveness to infection at this juncture, resulting in an exceedingly high fatality rate among these sepsis patients. The signaling pathways, inflammatory cytokines, recruited immunocytes, immune paralysis and complement system mentioned below are all closely involved in the cascade of inflammatory reactions in sepsis and its associated lung injury (**Figure 2**).

### 4.1 Inflammatory cytokine

The progression of sepsis is significantly influenced by the involvement of crucial cytokines such as IL-1β, IL-18, IL-6, IL-12, IL-17, and others. Il-1β is a member of the IL-1 family and is produced by inflammasome activation. Studies have shown that the level of IL-1β in non-survivors is higher than that in survivors, indicating that the high level of IL-1β is probably related to the adverse outcome of sepsis (156). The key pathological alteration contributing to the progression of sepsis to septic ARDS is the progressive increase in pulmonary vascular permeability. IL-1β has been found to increase endothelial permeability by inhibiting the transcription of VE-cadherin (157), suggesting that IL-1β is an important cytokine in the pathogenesis of ARDS induced by sepsis. IL-18 in patients with septic ARDS were significantly higher than patients with sepsis alone, demonstrating that IL-18 in sepsis are key cytokines causing lung injury (158). In a randomized controlled trial, plasma IL-18 levels were also discovered to be associated with the mortality in sepsis-induced ARDS (159). In studies of cytokines, IL-6 has also been suggested to correlate with the severity of sepsis (160, 161). IL-6 was frequently found in the blood and lung of patients with sepsis, but the exact role of IL-6 in sepsis is still under investigation, which may be related to the activation of the complement pathway and capillary leakage (162, 163). In addition, IL-12, IL-17, IFN-γ, and TNF-α all play important pro-inflammatory roles during sepsis. They interact and promote each other, eventually inducing inevitable organ damage. For example, during sepsis, dendritic cells, macrophages, and lymphocytes could secrete IL-12, one cytokine able to promote T cell differentiation. The production of TH1 and TH17 cells promote the secretion of IFN-γ and IL-17 (164). As the core cytokine in the cytokine storm, IL-17 can stimulate endothelial cells, epithelial cells, fibroblasts or macrophages to synthesize and secrete GM-CSF, IL-1β, IL-6, TNF-α and other cytokines and chemokines (165). The findings suggest that inflammatory cytokines play a crucial role as mediators in the development of lung injury induced by sepsis, and they synergistically interact to facilitate the progression of inflammation.

### 4.2 Inflammatory signal pathways

PRR can interact with PAMP and DAMP, activating the myeloid differentiation primary response protein 88 (MyD88) dependent pathway or Toll/IL-1R domain-containing adaptor-inducing IFN-β (TRIF) dependent pathway (166). Then, through this signal transduction, downstream nuclear factor κB (NF-κB) (167, 168), JAK/STAT (169, 170) mitogen-activated protein kinase (MAPK) (171–173) and other signaling pathways (174) are activated. These pathways upregulate the transcription of genes associated with inflammation, leading to the synthesis and release of various inflammatory molecules.

Some signal pathways in sepsis can cause lung injury and thus affect the disease process of ARDS. The NF-κB signaling pathway and its subsequent activation of NLRP3 inflammasome may be an important immune mechanism for ARDS induced by sepsis. In a sepsis induced ARDS rat mode, after the use of anti-TLR4 monoclonal antibodies, down-regulating expression of TLR4, MyD88, and NF-κB in macrophages and its alleviated pulmonary inflammatory injury were observed (168). NF-κB signaling pathway can further contribute to inflammasome production by activating the transcription of inflammasome components, such as the NLRP3 and the pro forms of cytokines (IL-18, IL-1β) (175). Blocking the activation of NLRP3 inflammasome and inhibiting the signaling pathway of NLRP3 can alleviate sepsis-induced lung injury (176).

The mitogen-activated protein kinases (MAPKs) pathway owns three major subfamilies, including the extracellular signal-regulated protein kinase (ERK) cascade, c-jun NH2 terminal kinase/stress-activated protein kinase (JNK/SAPK) cascade and p38-MAPK cascade (177). Multiple studies have found that ALI in sepsis patients can be significantly alleviated by inhibiting signaling pathways such as JNK and p38 MAPK (172, 178, 179). Pre-injection of specific inhibitors of JNK and p38 MAPK could decrease postoperative lung permeability and alleviate systemic inflammation after CLP inducing sepsis (172). One study on sepsis-induced ARDS also found that by inhibiting the activation of ERK1/2, p38 MAPK and p65, inflammatory infiltration and wet/dry ratio of lung tissue were all significantly decreased (171). Above all, pathways of ERK1/2, p38MAPK and JNK are probably involved in the lung injury induced by sepsis.

The activation of JAK/STAT signaling pathway has a dual effect on ARDS induced by sepsis. On the one hand, methotrexate (MTX), one inhibitor of the JAK/STAT signaling pathway, was shown to significantly reduce the production of pro-inflammatory cytokines and improve pulmonary inflammatory infiltration (169). Hence, inhibiting JAK/STAT signaling pathway could alleviate inflammatory lung injury in sepsis. On the other hand, several researchers have performed genome-wide analyses associated with ALI, the data suggest that the activation of STAT3 in type II alveolar pneumocytes (AT2) plays a dominant role in epithelial repair (180). Due to the bidirectional nature of this pathway, when targeting this pathway for anti-inflammation, it may be necessary to closely monitor the impact on epithelial repair when targeting this pathway for anti-inflammatory purposes.

### 4.3 Immune cells involvement

Sepsis-related circulating inflammatory overload causes pulmonary capillary endothelial damage and lung inflammatory injury, which involves a variety of immune cells. In the early stage of sepsis-induced lung injury, the massive release of pro-inflammatory cytokines results in disruption of vascular endothelial structures and endothelial leakage. Neutrophils and monocytes migrate across the endothelial cells and infiltrate in the alveoli (166).

Neutrophils are important components of innate immunity. There are four main mechanisms facilitating neutrophils fighting against microbial infection: degranulation, phagocytosis, cytokine production and NET. Neutrophil activation in the alveolar space is a universal feature of ALI in humans and animal models. During an episode of sepsis, circulating neutrophils undergo deformation, leading to their entrapment in the small capillaries of the pulmonary microcirculation. Trapped neutrophils respond to local chemokine gradients and migrate into lung tissue to cause lung injury (181).Neutrophils infiltrated in lung tissue can play a bactericidal and phagocytic role, simultaneously, their own secretion of cytotoxic products and the production of NETs can also lead to alveolar epithelial and endothelial damage (182, 183). In sepsis, the network structure of NET can limit the transmission and proliferation of pathogens, and the important components of NET such as histone, neutrophil elastase, and MPO can play a bactericidal role (184). NET has a definite role in controlling infection, but increasingly more studies have shown that excessive production of NET can cause thrombosis and tissue damage (182, 183, 185). In sepsis- induced ALI, activated platelets can promote the formation of tissue factor-rich NETs, which in turn can release thrombotic signals (182). Moreover, data from critically ill patients with sepsis and lung injury suggest that higher levels of circulating NETs will have adverse clinical consequences and even cause organ dysfunction (186, 187). For the moment, researchers tend to explore effective targeted drugs to inhibit NETs, so as to reduce multiple organ function damage and lung injury caused by sepsis (188–190).

During sepsis, alveolar macrophages are activated and they are able to secrete inflammatory cytokines to affect inflammatory environment. Alveolar macrophages can promote the trans-endothelial migration of neutrophils through the Src kinase/NAPDH oxidase pathway (191). Alveolar macrophages are essential for the maintenance of inflammatory homeostasis in the lung, and the impairment of 11β-hydroxysteroid dehydrogenase type-1(HSD-1) in alveolar macrophages leads to the aggravation of inflammation and the increased mortality in sepsis-induced ARDS (192). IFN-β can improve the dysfunction of alveolar macrophages in sepsis and reduce the mortality of septic ARDS (193). The mode of macrophage death can also affect lung inflammation, such as, pyroptosis. The up-regulation of IL-1R1 signaling caused by IL-1β can promote the pyroptosis of alveolar macrophages and aggravate lung injury (100).

Apart from that, lymphocytes and monocytes may also perform a crucial role in sepsis-induced ARDS. Single-cell sequencing of pneumonia, sepsis, and sepsis with ARDS found that the monocyte cluster in patients with early ARDS caused by sepsis was characterized by down-regulation of SOCS3 expression and up-regulation of multiple type I IFN-induced genes (194). In a bioinformatics analysis of key genes in sepsis-induced ARDS, compared with patients with sepsis alone, patients with septic-ARDS had higher levels of activated memory CD4^+^T lymphocytes and naive B lymphocytes, and lower levels of CD8^+^ T lymphocytes in their lung tissues (195). The findings of these studies suggest that lymphocytes and monocytes may play a pivotal role in the pathogenesis of this disease, necessitating further research.

### 4.4 Immune paralysis

The development and coexistence of systemic inflammatory response syndrome and compensatory anti-inflammatory response syndrome in sepsis exert a profound influence on the systemic immune environment (196).Initially, basic and clinical research on sepsis mainly focused on its excessive inflammation. But in contrast to the beneficial results of animal experiments, the clinical trials often failed, in which the management of inflammatory control and immunosuppressive drugs had been used (197–199). Due to the continuous development of immune paralysis in the process of sepsis progression, the use of immunosuppressive therapy may lead to treatment failure or even condition aggravation. The main mechanisms of septic immune paralysis include abnormal immune cell death, lymphocyte exhaustion, inhibition of antigen presentation, and expansion and activation of regulatory immune cells (200). When the apoptosis of immune cells is inhibited, the mortality rate of sepsis will decrease (201). Immune paralysis may also play an important role in the development of septic ARDS, and severe immunosuppression is often associated with adverse clinical outcomes. In one study, emergent lung and spleen harvest from patients who had died of sepsis revealed an expanded population of immunosuppressive cells in both organs and expression of inhibitory receptor ligands PD-L1 on lung epithelial cells, with extensive depletion of splenic CD4^+^, CD8^+^T cells and HLA-DR cells (202). After patients with sepsis were cured and discharged, systemic organs were still in a state of immunosuppression for a long time, and such patients were more likely to have secondary severe pulmonary infection (203).The suppression of pulmonary innate immunity induced by sepsis is mediated by IRAK-M molecules. When IRAK-M is knockout, septic mice would have a higher survival rate and bacterial clearance rate in the lung and blood (204). Immune paralysis dominates the systemic immune system in the later stages of sepsis, which contributes to the failure of immunosuppressive therapy. This compromised immune response renders patients vulnerable to infections and leads to a dismal prognosis, necessitating careful attention during the late-stage treatment process of septic ARDS.

### 4.5 Other mechanisms

The dysregulated systemic inflammatory response to infection can result in organ dysfunction throughout the body, leading to sepsis. Researchers found experimentally that complement is overactivated in septic hosts with adverse consequences (205–207). Complement activation is an innate immune response to fight infection, but excessive activation ultimately enhances organ dysfunction, including the heart, lungs and kidney (208–212). In sepsis, activation of the complement system is caused by recognition of PAMPS by mannose-binding lectins and ficolin, among others (213). DAMPs, the endogenous molecules released by distressed or damaged cell, can also lead to complement activation (213). The imbalanced activation of complement in the whole body can give rise to thrombotic inflammation and eventually bring about organ dysfunction. In sepsis-induced lung injury, complement activation can lead to the release of C3a and C5a, which can up-regulate the expression of endothelial adhesion molecules and recruit neutrophils to the alveoli (214). The released inflammatory molecules will recruit more inflammatory cells, eventually resulting in tissue damage and lung dysfunction (214). A marked increase in circulating complement activation products has been found in sepsis (215), and complement activation product C3a is associated with enhanced alveolar capillaries permeability (216). In addition, sepsis patients with enhanced capillary permeability are more likely to develop ARDS (217). The terminal complement complex exhibited an average increase of 110% two days prior to the progression from sepsis to ARDS, as demonstrated by a study (218). The above indicate that complement is closely related to the progression of sepsis to ARDS. The targeting of complement as a therapeutic approach for sepsis-induced lung injury had been investigated in several studies, yielding favorable outcomes (219, 220).

And beyond that, the coagulation and complement systems are closely related, and activation of the coagulation system is considered as part of the initial immune response to invading pathogens. Inflammation and coagulation are key host responses to infection and injury and have been implicated in the pathogenesis of both sepsis and ARDS (221). There is evidence that thrombosis and coagulation dysfunction are the main causes of ALI and ARDS (222). Both sepsis and ARDS are in a procoagulant state, and the interaction between the inflammatory response and the coagulation system plays a crucial role in the pathogenesis of sepsis-induced ARDS (223, 224). Sepsis can cause the alveolar-capillary barrier injury and further lead to the continuous state of promoting coagulation and fibrinolysis inhibition systemically (221, 225). In this process, tissue factor (TF) is considered to be the primary procoagulant initiation factor following infection and inflammation (226). Endotoxins or bacteria can activate TF-dependent clotting pathways to form a procoagulant state in the blood vessels and alveolar spaces of the lung (224, 227). TF can activate clotting factor VII, which further activates the downstream clotting cascade to produce thrombin. While thrombin causes fibrin deposition in interstitial spaces, perivascular spaces and pulmonary alveolus, high levels of thrombin may up-regulate lung inflammation genes and cause alveolar-capillary barrier damage (223). When subjected to pro-inflammatory stimulation (such as cytokine stimulation), alveolar epithelial cells will release TF-bearing microparticles, and the increase of circulating TF and microparticles expression levels will promote the coagulation cascade, resulting in the formation of thrombin and extensive fibrin inundation (228). Extensive pulmonary microthrombosis will increase dead space ventilation, which leads to the mismatch of ventilation-perfusion and causes respiratory dysfunction. An animal study on sepsis-induced ALI demonstrated that inhibiting the exogenous clotting pathway through inactivation of active site of FVIIa caused reduced fibrin deposition, decreased systemic cytokine response, and improved lung inflammation (229, 230). Nebulization of recombinant human-activated protein C(rh-APC) or plasma-derived human antithrombin had been found to attenuate lung coagulation activation, stimulate pulmonary fibrinolysis and alleviate lung inflammation without affecting blood clotting systemically (231). Nebulization of heparin or danaparoid also decreased pulmonary coagulopathy; however, they possess the drawback of impacting systemic coagulation (231, 232).

Researchers had found that thrombin-activated platelets and TF-enriched NETs facilitates immune thrombosis in sepsis-related lung injury, in which the two-hit procedures of thrombosis formation triggered by activated platelets and NETs contribute to the progression of ARDS (182). Besides, the expression of TF in peripheral blood monocytes induces intravascular thrombosis during sepsis as well (233).

## 5 Precision medicine of ARDS

Precision medicine is a medical strategy requiring clinicians to take into account the individual characteristics of patients, in which the patients will be grouped according to their heterogeneity, and receive targeted prevention, diagnosis and treatment. Numerous therapeutic approaches have garnered clinical attention for the treatment of ARDS, encompassing drug therapy, respiratory management, ECMO, and cell therapy. The utilization of inhaled surfactant therapy was initially employed in the neonatal respiratory distress syndrome (NRDS) due to the incomplete development of type II alveolar cells, which resulted in insufficient production of functional surfactant (234). Given the similarities between adult ARDS and NRDS, certain studies had also been conducted on inhaled surfactants for adult ARDS (235, 236). Although a multicenter randomized controlled trial in 1996 showed that inhaled surfactant did not improve 30-day mortality in sepsis-associated ARDS (237), recent studies in the treatment of COVID-19 have shown that surfactant administration can combat pulmonary dysfunction and enhance the diffusion of other drugs along the airway epithelium, which sheds new light on the application of surfactants (238, 239).

The inhibition of neutrophil elastase (NE) is crucial in mitigating lung injury caused by neutrophils, thus making NE inhibitors indispensable for the treatment of ARDS. In the past two years, the COVID-19 pandemic has brought renewed attention to the therapeutic role of NE inhibitors, which are anticipated to mitigate extracellular matrix degradation caused by neutrophils and restrict viral dissemination by inhibiting proteolytic activation of the S protein (240, 241). Compared with healthy individuals, the plasma level of NE in ARDS patients is significantly higher, and studies have found that SIRS patients with NE content over 220ng/ml are prone to ARDS (242). Elevated NE activity was also observed in bronchoalveolar lavage fluid of ARDS patients (243). The inhalation of prostaglandins has the potential to induce pulmonary vasodilation, decrease pulmonary artery pressure, and enhance oxygenation; however, there is substantial evidence suggesting that prostaglandins may elicit severe adverse reactions, such as hypotension (244).

The antifungal agent ketoconazole exhibits anti-inflammatory properties. Previous clinical studies have demonstrated its ability to prevent the progression of severe patients to ARDS (245), while showing limited efficacy in treating early-stage ARDS (246). The administration of ibuprofen did not demonstrate efficacy in preventing the development of ARDS in critically ill patients with sepsis (247); however, its early utilization for COVID-19 treatment has exhibited a preventive effect on complications and an improvement in prognosis (248).

ECMO is a form of extracorporeal life support technology, serving as an alternative therapeutic approach in cases of critical cardiac or pulmonary dysfunction (249). The device extracts blood, facilitates gas exchange outside the body, and then reinfuses the oxygenated blood back into the circulatory system of patients. Since the 2009 influenza A (H1N1) pandemic, ECMO has been documented to significantly reduce mortality in severe ARDS cases, thus garnering considerable attention and advancement (250–252). The utilization of ECMO in adults with severe ARDS was shown a significant reduction in 60-day mortality when compared to conventional mechanical ventilation (253). But a randomized controlled study demonstrated that there is no survival benefit associated with the early use of ECMO in severe ARDS when compared to its use as a rescue therapy after conventional mechanical ventilation (254). The use of ECMO is indicated for the treatment of severe ARDS following failure of protective mechanical ventilation and prone position ventilation (249, 255). Additionally, it can be employed in cases where the patient’s condition is unstable and urgent transfer to a specialized ward (256). ECMO has been extensively employed during the COVID-19 pandemic and showed significant improvements in patient outcomes and reductions in mortality rates across multiple studies (257, 258). Although ECMO has demonstrated survival benefits in several studies of severe ARDS, it still entails numerous risks, including infection (259) and coagulation related complications (257, 260). Therefore, careful attention must be paid to its indications and contraindications during ECMO utilization to reduce mortality.

Mesenchymal stem cells and their extracellular vesicles have been shown to modulate immunity and improve endothelial barrier integrity in cellular and animal experimental studies of ARDS (261, 262). However, in the recent randomized controlled trials of severe ARDS from COVID-19, no benefit was found on survival and ventilator-free days (263). Whether mesenchymal stem cell-related cell therapy is suitable for ARDS, a rapidly progressive disease, needs to be further studied and explored.

A randomized controlled trial found that only those patients with severe ARDS (PaO2/FiO2 ≤ 150mmHg) had a good response to prone position ventilation. And prone position ventilation significantly reduced 28-day and 90-day mortality in these patients (24). In ARDS caused by COVID-19, the use of dexamethasone combined with respiratory support can reduce the mortality of patients (264). A randomized controlled trial reported in 2020 claimed that the early use of dexamethasone reduced the duration of mechanical ventilation and mortality in patients with moderate and severe ARDS (265). Therefore, dexamethasone may be a good choice for patients with moderate-to-severe ARDS or patients with COVID-19 who have extensive lung injuries. In addition, in a randomized controlled trial, there was no significant difference between the simvastatin treated and the placebo-treated in the trial of ARDS. But in a secondary analysis of this study, patients were grouped according to inflammatory degrees, and the therapeutic effect of simvastatin on the hyperinflammatory phenotype was observed (27). The common treatments for ARDS are lung-protective ventilation, prone position ventilation, and fluid therapy. According to the heterogeneity of the disease, clinicians would not blindly use these methods for all patients without subgrouping, but take the individual characteristics into consideration to implement targeted and effective treatment.

In view of the particularity and risk of sepsis-induced ARDS, advanced treatment methods are expected to be developed urgently. Inflammatory infiltration and high vascular permeability are the key factors of sepsis-induced ARDS (266), which are two important targets worth cutting into. For example, in the aspect of inflammation, the inhibition of protein expression in key inflammatory signaling pathways and anti-complement antibodies are used to control septic ARDS in animals (171, 267, 268). In terms of vascular endothelial permeability, protection by endothelial connexin is enhanced by inhibiting proteins that affect the endothelial barrier or natural endothelial-protective factors (266, 269). In the study of sepsis patients, the levels of plasma Ang-2 were found to be associated with ANGRT2 genetic variants, which would lead to an increased risk of ARDS (270). For patients with high plasma ANG-2 levels, lowering ANG-2 levels may be an appropriate strategy for the prevention and treatment of sepsis-induced ARDS.

## 6 Conclusion and prospect

ARDS is a heterogeneous syndrome, and different etiology, severity of symptoms, and individual genetic differences can bring about various clinical manifestations and responses to treatment. In view of the heterogeneity of ARDS, increasingly more researchers and clinical staff are inclined to classify ARDS, and further carry out personalized treatment to obtain beneficial results. For example, in studies using statins and prone ventilation to treat ARDS, patients with severe ARDS (PaO2/FiO2 < 150mmHg) were specifically selected for these two trials, and treatment-related survival benefits were obtained (24, 271). Given the heterogeneity of ARDS, categorizing patients with ARDS into pulmonary and extrapulmonary groups based on the etiology is another solution having received much attention. There are differences between pulmonary ARDS and extrapulmonary ARDS in pathological manifestations, imaging findings, respiratory mechanics, mortality, biomarkers and even genetics (32, 43, 63, 72, 272).

Sepsis is a systemic disease in which the immune system reacts out of control in response to infection or injury, leading to systemic organ dysfunction. When sepsis progresses to the advanced stage of shock, it is more likely to be accompanied by ARDS and higher mortality rate (273). Sepsis-induced lung injury primarily arises from vascular endothelial damage, leading to the transmission of circulating inflammation to the lungs. This condition predominantly manifests as damage to the lung capillary endothelium and alveolar epithelium, cell death, oxidative stress, microcirculatory disorders and pulmonary edema. Pathological manifestations of that disease include diffuse alveolar damage, destruction of alveolar capillaries, pulmonary edema, and potentially even atelectasis (91).

The immune mechanism of sepsis-induced ARDS is mostly related to the pathogenesis of sepsis itself, involving excessive activation of inflammatory signaling pathways and cytokines, activation of immune cells, immune paralysis and interaction of coagulation and complement. The cascade of cytokine activation in sepsis is able to cause systemic inflammation, including the lungs. Sepsis causes lung inflammation and injury by damaging the vascular endothelium, and the activation of endothelial cells give rise to the accumulation of inflammatory cells and inflammatory cytokines secretion. The aggregation of neutrophils can produce a large amount of myeloperoxidase and NETs, and then cause vascular thrombosis. Alveolar macrophages can regulate lung inflammation, and most of them can promote the amplification of inflammatory response and increase the concentration of cytokines. The death and apoptosis of alveolar macrophages can promote the accumulation of neutrophils, and their pyroptosis process also produces inflammatory cytokines. Obvious immune paralysis occurs in the later stage of sepsis, which often indicates poor prognosis and high mortality (274). The immunosuppression of sepsis is similar to that of tumors, which is related to the inhibition of T lymphocytes caused by PD-1 pathway. When immune paralysis is relieved, the prognosis of patients can be greatly improved. Perhaps, PD-1 and PD-L1 can also become important targets of septic-ARDS. After being discharged, surviving patients would undergo longer periods of immunosuppression and are more likely to lack of resistance against infection (202, 203).

At present, the solution to the heterogeneity of ARDS is to pay attention to the manifestations and biological subgroups of ARDS, such as PaO_2_/FiO_2_ level and inflammation degree, and give candidate therapeutic strategies according to the classification. The heterogeneity of ARDS affects the efficacy of various treatments, including drug therapy, respiratory management, ECMO (275). The discovery of the heterogeneity of ARDS had benefited from the latent class analysis of randomized controlled trials, which has made advances in the treatment of specific subgroups. There are still existing many unanswered questions regarding the advancement of precision medicine in ARDS, such as the biologic overlap between ARDS and sepsis, the impact on patient recruitment, the stability of ARDS subphenotypes and so on (276). The resolution of key issues can promote the progress of effective treatment of ARDS and usher in the progress of severe disease research.

According to a report from the Lancet Respiratory Medicine in 2022, precision medicine for ARDS is necessary, but there are still two major challenges (277). One is that the key nodes have limited information and cannot be determined. The pathogenesis of ARDS is complex, and the specific pathways involved will become deranged during the disease process. The other is rapid disease progression of ARDS, with a high mortality rate within 72 hours (278). At present, precision medicine is widely used in the field of cancer, but it still takes days to weeks to achieve staging and classification. The time requirements in the precision management of ARDS are deemed impractical, and it is crucial to rapidly determine our precise classification of the disease within a matter of hours.

## Author contributions

HX: Writing – original draft, Writing – review & editing. SS: Writing – original draft, Writing – review & editing. WL: Conceptualization, Writing – review & editing. XX: Writing – review & editing. ZZ: Conceptualization, Funding acquisition, Validation, Writing – review & editing.
